# Incidence of chronic subdural haematoma: a single-centre exploration of the effects of an ageing population with a review of the literature

**DOI:** 10.1007/s00701-021-04879-z

**Published:** 2021-06-28

**Authors:** D. J. Stubbs, M. E. Vivian, B. M. Davies, A. Ercole, R. Burnstein, A. J. Joannides

**Affiliations:** 1 University Division of Anaesthesia, Department of Medicine, Addenbrooke’s Hospital, Hills Road, Cambridge, CB2 0QQ UK; 2 Department of Clinical Neurosciences, University of Cambridge, Cambridge, CB2 0QQ UK

**Keywords:** Chronic subdural haematoma, Epidemiology, Incidence, Health service planning

## Abstract

**Background:**

Chronic subdural haematoma (cSDH) is a common neurosurgical pathology frequently occurring in older patients. The impact of population ageing on cSDH caseload has not been examined, despite relevance for health system planning.

**Methods:**

This is a single-centre study from the UK. Operated cases of cSDH (*n* = *446*) for 2015–2018 were identified. Crude and directly standardised incidence rates were calculated. Medline and EMBASE were systematically searched to identify studies reporting on the incidence of cSDH by year, so an estimate of rate of incidence change could be determined. Local incidence rates were then applied to population projections for local catchment area to estimate operated cSDH numbers at 5 yearly intervals due to shifting demographics.

**Results:**

We identified nine studies presenting incidence estimates. Crude estimates for operative cases ranged from 1.3/100,000/year (1.4–2.2) to 5.3/100,000/year (4.3–6.6). When non-operated cases were included, incidence was higher: 8.2/100,000/year (6.0–11.2) to 48/100,000/year (37.7–61.1). Four pairs of studies demonstrated incidence rate increases of 200–600% over the last 50 years, but data was deemed too heterogeneous to generate formal estimate of incidence change. Local crude incidence of operated cSDH was 3.50/100,000/year (3.19–3.85). Directly standardised incidence was 1.58/100,000/year (1.26–1.90). After applying local incidence rates to population projections, case numbers were predicted to increase by 53% over the next 20 years.

**Conclusions:**

The incidence of cSDH is increasing. We project a 53% increase in operative caseload within our region by 2040. These are important findings for guiding future healthcare planning.

**Supplementary Information:**

The online version contains supplementary material available at 10.1007/s00701-021-04879-z.

## Introduction

Chronic subdural haematoma (cSDH) is a common neurosurgical condition, consisting of a collection of altered blood lying beneath the dural membrane. Although widely thought of as the result of a progression of an acute subdural bleed, significant numbers occur after no significant antecedent trauma, perhaps due to an inflammatory process occurring at the level of the dural border cell [9]. cSDH is widely considered a disease of old age [28] with significant levels of comorbidity and polypharmacy present amongst those referred for surgery [7].

The demography of the worldwide and UK population is changing due to a combination of falling fertility and mortality rates, and by 2039, it is estimated that the number of individuals aged over 60 will increase from 14.9 to 21.9 million [12]. Such a change in population structure will lead to rising numbers of conditions associated with older age [12]. In single-centre studies examining traumatic brain injury (TBI) in the older patient, over 37% of cases occurred in those over 65, of which a third were due to SDH (both acute and chronic) [25]. In the context of rising admissions for TBI in older adults (39 million emergency department visits in 2009–2010 in the USA) [18], the number of patients presenting for cSDH surgery is likely to increase substantially.

Patients presenting with cSDH will require contact with neurosurgical centres for decisions regarding surgery, the operation itself, and immediate postoperative care. Such a ‘hub and spoke’ transfer system has the potential to create a bottleneck in the care of this vulnerable patient cohort. A 2007 audit of neurosurgical bed pressures [8] identified that nearly 30% of all patient bed days were due to delays in either surgery, inter-hospital transfer, or radiological investigation. The vast majority (92%) of these delays occurred in emergency neurosurgical patients [8].

In this study, our main aim is to examine how projected demographic shifts may change the number of cases of cSDH that may present for surgery at our centre and discuss the potential impact. To inform our modelling, we first systematically searched the literature to identify whether an estimate of underlying change in incidence rate could be incorporated into our modelling. Following this, we present our local incidence rate of operated cSDH and, using population projections from the office for national statistics (ONS), estimate the projected change in operative workload that could arise due to demographic shifts.

## Methods

### Setting

The study centre provides neurosurgical services to hospitals across the East of England, a population of approximately 5.8million in 2011 [17]. Services are provided 24 h a day, 365 days a year. An electronic database of referrals is maintained by the on-call neurosurgical service. Broadly, patients can be considered referred from an external hospital (to which they will be repatriated after completion of surgery) or from within the study centre (for patients for whom this is their local secondary care centre).

### Approvals

Aggregate case numbers by age were obtained as part of an institutionally approved retrospective evaluation of service as permitted in UK practice (reference PRN7703).

### Systematic literature search

To provide important context to our findings and inform our modelling approach we systematically identified other studies examining the population incidence of cSDH. Medline and EMBASE were searched up to the 22^nd^ April 2020 to identify papers publishing incidence estimates of cSDH. Keywords to identify cSDH were combined with phrases to identify epidemiological or incidence studies. Subject headers were used where appropriate. Full search strategy is shown in Supplementary material. Title and abstract screening as well as data extraction was conducted by 2 authors (DJS and MEV) with full-text inclusion based on a priori determined inclusion criteria (Supplementary Material Table S2). Where required, incidence values were calculated using supplied case and population data in each paper and confidence intervals calculated using normal approximations. All incidences are expressed as *n/100,000/year.*

### Regional incidence of operated cSDH: case and population definitions

For this study, cases that underwent surgery for cSDH were identified in the following manner. Cases were identified from the neurosurgical referrals database and cross-referenced against theatre activity data. This is a more robust methodology for case ascertainment, given international classification of disease (ICD) codes for subdural haematoma are not specific [19]. All cases between 2015 and 2018 (for which whole year information was available) were included. Referrals are received from hospitals close to the London boundary where neighbouring neurosurgical centres are located. As such it is conceivable that a proportion of cases may have been referred to another centre and thus be missing from our records. To minimise this, we only included cases referred from hospitals located within Cambridgeshire, Suffolk, Bedfordshire, and Norfolk to allow a more accurate estimate. The population at risk was defined using the 2016 population covered by these clinical commissioning groups as published by the Office for National Statistics [ONS], UK [23].

### Projection of case numbers due to an ageing population

Calculated regional incidence rates for operated cSDH were applied to population projections for the same region as defined by the ONS [23]. This was done for the years 2025–2040 at 5-year intervals. Upper and lower boundaries of this estimate were calculated using the limits of the 95% confidence interval for our calculated regional rate between 2015 and 2018.

### Statistics and direct standardisation

All analysis was conducted using the statistical software R (v 3.5.3) [20]. Confidence intervals are Poisson exact, and all incidences are expressed per 100,000 individuals at risk per year. Where necessary, 95% confidence intervals [95% CI] were calculated as normal approximations using the standard error of the natural logarithm of the incidence rate (*I*).

Direct standardisation of incidence rates was performed to the World Health Organization (WHO) 2000–2025 world standard population [27]. Details of these calculations are shown in the Supplementary material.

## Results

### Systematic literature search

**Fig. 1:**
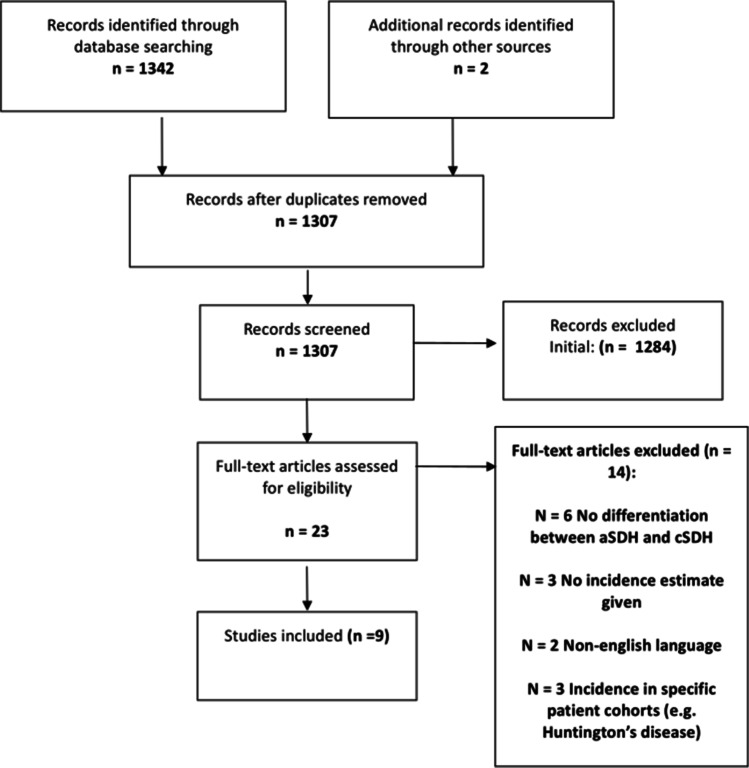
Article inclusion in systematic literature search for incidence estimates of chronic subdural haematoma

Literature searching identified 1342 articles with two further articles identified from the references of these papers, one of which was included and one which was excluded (published in Japanese). In total, nine papers were screened for details of published incidence rates. Details of inclusions and exclusions are shown in Fig. 1.

Eight studies presented crude incidence rates for their population as a whole (Table 1) [2, 4, 10, 13–15, 21, 22]. Incidence ranged from 1.7 in the period 1967–1973 in Finland [10] to 48 in 2017 in North Wales [2]. For two studies [14, 22], incidence rate was calculated from presented case numbers. In one of these [22], estimated total population was calculated as the midpoint between quoted values for the beginning and end of the study period. Case ascertainment differed between studies, with earlier studies reporting on only operated or autopsy identified cSDH [10]. For instance, in the Mellergard study, they point out that in their institution, routine CT scanning for diagnosis did not become widespread until 1985 [15]. Studies which only included operated cases all report lower incidences compared to those including radiological diagnoses (Table 1). Between the two time points studied in one paper (1969 and 1983), there was a 250% increase in reported crude incidence of cSDH [15]. This is matched by a repeated study within North Wales which demonstrated an increase in incidence in those over 65 from 8.2 to 48 between 1999 and 2017[1] as well as studies from both Finland [21] and Brazil [14] where data was available over a 25- and 8-year period, respectively.

**Table 1:** Published crude estimates of the incidence (per 100,000/year) of chronic subdural haematoma identified through systematic literature search

Author	Year	Location	Incidence (/100,000/year)	Type	N	95%CI
Fogelholm [10]	1967–1973	Helsinki, Finland	1.7	Operated and autopsy	64	1.4–2.2
Mellergard [15]	1969	Lund, Sweden	2.0	Operated	28	1.4–2.9
Mellergard [15]	1983	Lund, Sweden	5.3	Operated	82	4.3–6.6
Kudo [13]	1988	Awaji Island, Japan	13.1	All	66	10.3–16.7
Asghar [4]	1997–1999	North Wales	8.2	All	40	6.0–11.2
Adhiyaman [1] *	2017	North Wales	48.0	All	66	37.7–61.1
Rust [22]	2006	Tasmania	3.5	Operated	81	2.8–4.4
Rauhala [21]	1990–1995	Pirkanmaa, Finland	8.2	All	167	7.0–9.5
Rauhala[21]	2010–2015	Pirkanmaa, Finland	17.6	All	354	15.9–19.5
Magalhães[14]	2008	Brazil	1.3	Hospital admissions	2389	1.2–1.3
Magalhães[14]	2015	Brazil	2.4	Hospital admissions	4885	2.3–2.4

*Indicates that incidence was calculated in those aged 65 or older only. Magalhães [14] used a hospital admission authorisation code. Unclear if captured non-operated hospital admissions. *95% CI* 95% confidence intervals calculated for all studies (for details, see “Methods”)

However, due to the paucity of studies, mixed methods of case ascertainment, and lack of direct standardisation, comparisons are difficult and thus no formal meta-analysis was performed.

Four studies reported incidence stratified by age and, in three cases, by gender as well. One study [6] only reported stratified incidence rates without a crude population estimate (Table 2).

**Table 2:** Age and sex-stratified incidence (per 100,000/year) from studies identified in systematic review of the incidence of chronic subdural haematoma

Study	Year	Location	Age range	Sex	Incidence	N	95% CI
Fogelholm [10]	1967–1973	Helsinki, Finland	20–29	M	0.25	1	0.0–1.8
				F	-	-	-
			30–39	M	1.56	4	0.6–4.2
				F	0.76	2	0.2–3.0
			40–49	M	4.0	8	2.0–8.0
				F	1.2	3	0.4–3.7
			50–59	M	9.1	15	5.5–15.1
				F	0.8	2	0.2–3.2
			60–69	M	8.4	10	4.5–15.6
				F	1.8	4	0.7–4.8
			70–79	M	15.7	7	7.5–32.9
				F	4.2	5	1.8–10.1
			80 +	M	-	-	-
				F	7.8	3	2.5–24.2
Kudo [13]	1992	Awaji Island, Japan	< 65	All	3.4	14	2.0–5.7
			65 +	All	58.1	52	44.3–76.3
Bartek [6]	2005–2010	Norway/Sweden	18–49	All	0.6	-	
			50–66	All	7.1	-	
			67–79	All	27.3	-	
			80–89	All	59.5	-	
			90 +	All	52.0	-	
Rauhala [21]	1990–1995	Pirkanmaa, Finland	18–59	M	3.3		
				F	1.2		
			60–69	M	22.6		
				F	6.2		
			70–79	M	61.9		
				F	16.5		
			80–89	M	96.4		
				F	33.3		
			90 +	M	56.6		
				F	0		
Rauhala [21]	2011–2015	Pirkanmaa, Finland	18–59	M	2.0		
				F	2.1		
			60–69	M	28.2		
				F	8.9		
			70–79	M	82.3		
				F	28.3		
			80–89	M	226.0		
				F	79.3		
			90 +	M	231.0		
				F	94.2		

*M* male, *F* female, *95%CI* 95% confidence interval, *n* number of cases. Confidence intervals calculated (see “Methods”) for Fogelholm and Kudo studies. No confidence intervals could be calculated for the Bartek study as the number of cases was not presented in the paper

The importance of change in population demographics was highlighted in a Finnish study which demonstrated a significant increase in incidence amongst older males between 1990 and 2015 [21]. Over this period, incidence in males aged over 90 increased from 56.6 to 231. The authors also report a doubling of the population aged over 80 over this time period [21].

### Regional incidence of operated cSDH in the East of England

Between 2015 and 2018, there were 446 cases of initial surgery for cSDH referred from hospitals in the area described. This reflects 86.1% of total surgery performed at CUH for cSDH over the same period. Over the studied period, the crude incidence of operated cSDH was 3.50 (3.19–3.85). The directly standardised incidence rate was 1.58 (1.26–1.90). The number of cases per year increased from 86 in 2015 to 114 in 2018. Calculated incidence rates by age are shown in Table 3.

**Table 3:** Incidence (per 100,000 per year) of operated cases of chronic subdural haematoma referred from Bedfordshire, Cambridgeshire, Norfolk, and Suffolk (2015–2018) *n* = *446* (*95% CI* 95% confidence interval, population at risk defined using Office for National statistics 2016 population projection for these counties. Confidence intervals are Poisson exact)

Age group	*N*	Incidence (/100,000/year)	95% CI
0–9	0	0.00	0.00–0.24
10–19	0	0.00	0.00–0.24
20–29	3	0.20	0.04–0.57
30–39	4	0.25	0.07–0.65
40–49	14	0.83	0.46–1.40
50–59	30	1.77	1.20–2.53
60–69	65	4.32	3.33–5.51
70–79	163	15.08	12.85–17.58
80–89	147	25.71	21.72–30.21
90 +	20	15.49	9.46–23.92

**Fig. 2:**
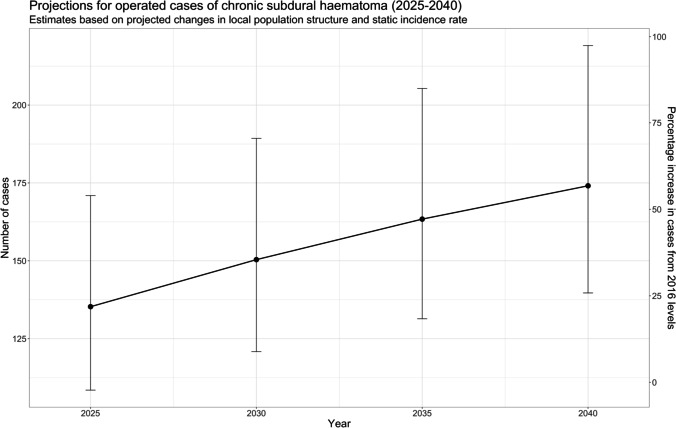
Projected number of operations for chronic subdural haematoma (cSDH) from our study region between 2025 and 2040. Calculations assume constant local incidence but changing population age structure based on office for national statistics population projections. Error bars demonstrate upper and lower bounds of estimates based on 95% confidence intervals for locally calculated incidence rate

Incidence rates were then applied to the ONS projections for the population in these counties between 2020 and 2040 at 5-year intervals (Table 4). Compared to the 114 referrals seen in 2014, these estimates would suggest a 52.6% rise in cases of operated cSDH by 2040. Assuming that these counties continued to represent 86% of all referrals for cSDH surgery, by that time, the actual number of operated cases could be 202 (163–255). This trend (extrapolated to all referrals) is shown in Fig. 2.

**Table 4:** Projected increase in number of operations for chronic subdural haematoma referred from Bedfordshire, Cambridgeshire, Norfolk, and Suffolk arising solely due to estimated changes in population age structure 2025–2040 (*95% CI* 95% confidence interval). Projections assume locally calculated incidence rates remain constant; hence this only models changes arising due to population age structure in the indicated year. Population projections are based on Office for National Statistics population projections for named counties. Confidence intervals are based on upper and lower estimates of calculated incidence rates (Table 3)

Year	Projected cases	95% CI
2025	135	108–171
2030	150	121–189
2035	163	131–205
2040	174	140–219

## Discussion

There are two key findings from our work. Firstly, that there is clear literature evidence of an underlying rise in the incidence of cSDH. Secondly, even assuming local incidence rate remains constant, we predict that population ageing alone could result in a 53% (23–92) increase in operations for cSDH by 2040 at our centre. Such findings and the medical complexity of this operative cohort mean that neurosurgical services will have to adapt to deal with such a rise.

Our local, crude incidence of operated cSDH of 3.5 is broadly compatible with other estimates derived from operative data (Table 1). The significance of these findings on neurosurgical services is clearly apparent. Patients with cSDH exhibit a significant degree of inpatient morbidity [24] that negatively impacts on length of stay. Any increase in this workload will further stress a system that already struggles to cope with the complexities of workload, capacity, referral and repatriation [8]. Given the interlinked nature of care for these medically complex patients, involving multiple hospitals and health systems, any rise in cases will require an integrated approach to ensure optimal care. Our systematic search strategy enables us to present an unbiased view of local incidence rates within the wider literature context. Two broad, unadjusted trends are apparent. Firstly, that incidence across all studies increased with age and a male preponderance exists, with incidence in the oldest males over threefold higher than in women (Table 2). The results in Table 1 could be interpreted as suggesting an increasing incidence over time. Due to the disparate geographic locations of the studies as well as differences in case ascertainment, the potential for confounding of this association is high. As such we did not attempt meta-regression or the incorporation of rising incidence rate into our projections. Some of this trend is almost certainly driven by increased rates of detection due to improved diagnostic technologies [15], especially when the rates in 1967–1973 [10] were based solely on operated or autopsy cases. However, studies where incidence was calculated at different time points in the same population appear to suggest a true rise [2, 14, 15, 21]. Although some of this could arguably be confounded by widespread adoption of medical imaging technology, this should not be a significant factor for the rise in case numbers seen over the last decade in Brazil [14] or the linearity in incidence rise over the last 20 years in Finland [21].

Being as published incidence figures are crude, it is possible that this apparent rise merely reflects the changing population demographics captured in each setting [21], but a distinct aetiological driver could exist. Certainly, other authors have highlighted the higher incidence of SDH in those taking anticoagulant and antiplatelet medications [11], whilst rising rates of prescription of direct oral anticoagulant (DOAC) drugs are temporally related to increased admissions for bleeding complications, including intracerebral haemorrhage [3].

Extrapolating from a single-centre study is hindered by a lack of external validity, and the magnitude of our findings cannot be directly applied to other centres. Although the broad trend of population ageing is well established [12], there will be a certain degree of heterogeneity in how this affects individual centres for instance based on their location and urban/rural split. Regardless, our methodology, literature examination, and broad trend will be of interest to the wider community. A study from the USA used similar methods to ours and estimated a growth in SDH cases of 53.3% between 2020 and 2060 [16]. Importantly, they used ICD codes for case ascertainment, and as such, these figures include cases of acute SDH (aSDH). Given the differences in case definition and population structure, it is difficult to compare the relative increase in cSDH cases directly, but regardless of this, as the authors highlight, this rise in workload is likely to outstrip projected workforce growth [16].

The key strengths and limitations of our study both revolve around case definition. This is a well-recognised problem in the literature, as ICD codes for traumatic and non-traumatic subdural haematoma have poor discriminative performance when compared to a clinical review of diagnostic imaging [19] and cannot take the spectrum of disease between acute and chronic subdural haematomas into account. As such, ‘contamination’ of the numerator in incidence calculations using these methods by cases of acute SDH is a possibility. By focusing on operated cSDH, we hope to have minimised this by using a clinician-coded database to identify cases (by diagnosis of cSDH) which was then cross-referenced against theatre activity data (where procedure codes are once again shared with operative interventions for aSDH). As well as seeking to refine our case definition, we also took steps to better define our population at risk. This was performed by excluding cases referred from the periphery of our region, removing patients referred from centres on the boundary between adjoining neurosurgical centres.

The impact of case definition on reported incidence is also apparent in the wider literature. We excluded several studies that did not take steps to differentiate between aSDH and cSDH. These represent two extremes of a spectrum of disease that partly explains the difficulties in case ascertainment using diagnostic codes. It is however true that aSDH more commonly affects younger patients, is likely to be associated with significant extra-cranial injuries, and mandates a distinct surgical approach [20]. One excluded paper [5] justified including acute SDH in their cases of ‘chronic’ SDH by explaining that many cSDH contain elements of acute blood (acute on chronic SDH). We chose to focus on purely chronic cases of cSDH for which surgical techniques (such as burr hole drainage) are well characterised and that can arise by both traumatic and non-traumatic means [41]. This was intentional, to reduce heterogeneity in our case definition and provide information to facilitate the planning of services caring for the medically complex cohort which this disease classically afflicts.

What our study cannot comment on is the true population burden of cSDH. This is likely to be substantially higher, a nationwide survey of UK practice suggested that just under 70% of cSDH referrals were ultimately accepted for surgery [7]. From the perspective of a neurosurgical centre, this unmeasured need means that we are unable to comment on the potential for growth in non-operative workload (e.g. referrals, advice, imaging) that could occur due to shifting population demographics. The integrity of our findings on operative workload however is not affected by this and is of vital importance for future capacity planning.

## Conclusions

There is the potential, over the next 2 decades, for a significant rise in operations and referrals for cSDH. This cohort is well recognised to be older and with significant comorbidity [7]. A retrospective review of emergency neurosurgical patients over 75 demonstrates significant rates of inpatient complication, institutional discharge, and length of stay [26]. Any projected rise in cases will necessitate either an increase in neurosurgical capacity (within existing centres or new facilities), improvements in efficiency, or both. Given the ‘hub and spoke’ nature of neurosurgical referral, any approach to catering for this rise in case numbers should arguably look at the entirety of the patient journey. Further work should focus on barriers to current patient care, refinement of models for projected case numbers, and modelling of the impact of any rise in case numbers on other aspects of neurosurgical and wider hospital care.

## Supplementary information

ESM 1 (DOCX 44 kb)

## Declarations

### Disclaimer

The views are those of the authors and not necessarily those of the NHS, the NIHR, or the Department of Health.
