# Supplementary material for: Incidence of chronic subdural haematoma: a single-centre exploration of the effects of an ageing population with a review of the literature
Source: Acta Neurochir (Wien). 2021 Jun 28;163(9):2629–37. doi: 10.1007/s00701-021-04879-z (PMC8357776; doi:10.1007/s00701-021-04879-z)
Supplement: Supplementary file 1 — (DOCX 44 kb) [file 701_2021_4879_MOESM1_ESM.docx]

## Supplementary Material:

## Incidence of chronic subdural haematoma: a single centre exploration of the effects of an ageing population with a review of the literature.

*Stubbs DJ, Vivian ME, Davies B, Ercole A, Burnstein R, Joannides A*

**Contents:**

1. **Systematic search strategy and inclusion/exclusion criteria**
2. **Incidence calculations and direct standardisation**
3. **Population projections**
4. **R Code for calculation of projected case numbers**
5. **Estimated case numbers for local region and EoE (output of R code in 4)**

**1: Search Strategy and inclusion/exclusion criteria for systematic search**

*(shown for Embase database – equivalent terms used in Medline).*

Database: Embase <1974 to 2019 March 22>

Search Strategy:

--------------------------------------------------------------------------------

1 exp epidemiology/ or epidemiology.mp. (3563491)

2 exp incidence/ or incidence.mp. (1099330)

3 or/1-2 (4042554)

4 chronic subdural h?ematoma.mp. or *subdural hematoma/ (7102)

5 3 and 4 (1044)

6 limit 5 to english language (929)

***************************

*Exp refers to inclusion of the term as a subject heading.*

| **Inclusion** | **Exclusion** |
| --- | --- |
| Cases of cSDH ascertained using surgical, radiological, pathological, orr diagnostic code criteria | aSDH cases or mixed (with no effort to select only cSDH) cases |
| Incidence must be expressed for a general and identified population | Secondary literature (e.g. reviews, editorials) |
| Cohort studies | Case-control studies |
| Cases can be derived from either hospital, community, or both | Published as abstract only |
|  | Paediatrics (<18 years of age) |
|  | Non- English language |

**Supplementary Table S1:** Inclusion and exclusion criteria used in systematic review of the incidence of chronic subdural haematoma (cSDH = chronic subdural haematoma, aSDH = acute subdural haematoma)

**2: Incidence and direct standardisation**

**Supplementary Table S3: Incidence and direct standardisation**

***N in standard population*** *= number at risk in WHO standard population*

***N of cases*** *= number of operated cases of cSDH by age bracket (M+F)*

***Cohort incidence*** *= incidence of operated cSDH expressed per 100,000person years or per year (py)*

***N in cohort population*** *= number at risk based on 2016 mid-year population estimates for Suffolk, Norfolk, Bedfordshire, Cambridgeshire*

***Ni^2.ri/ni =*** *Number in reference popln. Rate in ith stratum/n in ith stratum (to calculate variance of DSR)*

***95% upper and lower*** *= upper and lower bounds of 95% confidence intervals*

| **Age** | ***n in standard pop*** | ***n of cases*** | ***expected n*** | ***cohort incidence (per 100,000 person years)*** | ***cohort incidence (per person year)*** | ***n in cohort pop.*** | ***Ni^2.ri/ni*** |  | | |  |  |
| --- | --- | --- | --- | --- | --- | --- | --- | --- | --- | --- | --- | --- |
| 0-9 | 175,439 | 0 | 0 | 0 | 0 | 388044 | 0 |  |  |  |  |  |
| 10-19 | 170,640 | 0 | 0 | 0 | 0 | 351958 | 0 |  | | | **Crude incidence =** | 3.50491502 |
| 20-29 | 161,443 | 3 | 0.322886 | 0.2 | 0.000002 | 384027.4 | 0.13573949 |  | | | Ln(I) | 1.25416627 |
| 30-39 | 147,548 | 4 | 0.36887 | 0.25 | 0.0000025 | 394281.6 | 0.13803847 |  | | | SE(ln(I) | 0.04735137 |
| 40-49 | 126,256 | 14 | 1.0479248 | 0.83 | 0.0000083 | 419179.7 | 0.31563264 |  | | | N | 446 |
| 50-59 | 99,165 | 30 | 1.7552205 | 1.77 | 0.0000177 | 422692.1 | 0.41178068 |  | | | 95% Upper | 3.84577431 |
| 60-69 | 66,777 | 65 | 2.8847664 | 4.32 | 0.0000432 | 375553 | 0.51293971 |  | | | 95% Lower | 3.19426682 |
| 70-79 | 37,287 | 163 | 5.6228796 | 15.08 | 0.0001508 | 270287.4 | 0.77569399 |  | | |  |  |
| 80-89 | 13,495 | 147 | 3.4695645 | 25.71 | 0.0002571 | 142941 | 0.32756013 |  | | |  |  |
| 90+ | 1,950 | 20 | 0.302055 | 15.49 | 0.0001549 | 32282.69 | 0.0182453 |  | | |  |  |
| **Total:** | 1,000,000 | 446 | 15.7741668 |  |  | 3181246.89 | 2.63563041 |  | | |  |  |
|  |  | **DSR** | 1.57741668 |  | **Variance of DSR (v)** |  | 2.6356E-12 |  | | |  |  |
|  |  | **95% Lower** | 1.25921803 |  | **SE of DSR** |  | 1.6235E-06 |  | | |  |  |
|  |  | **95% Upper** | 1.89561533 |  |  |  |  |  | | |  |  |

| **Crude incidence =** | 3.50491502 |
| --- | --- |
| Ln(I) | 1.25416627 |
| SE(ln(I) | 0.04735137 |
| N | 446 |
| 95% Upper | 3.84577431 |
| 95% Lower | 3.19426682 |

**Supplementary table S4: Incidence confidence intervals**

***Crude incidence (/100,000 person years)***

***Ln(I) =*** *natural logarithm of the incidence rate*

***SE(ln(I))*** *= Standard error of the natural logarithm of the incidence rate*

***N =*** *Number of cases (Ln(I) = 1/*$\surd N$*)*

***95% Upper and Lower*** *= upper and lower confidence intervals of the calculated incidence rate*

**Calculation of confidence intervals**

Confidence intervals for the Directly Standardised Rate (DSR) were calculated as $DSR\pm1.96 . \surd v$ where:

$$v= \sum_{i=1}^{k} \frac{{N_{i}}^{2}r_{i}/n_{i}}{{\sum_{i=1}^{k} N_{i}}^{2}}$$

and,

*Ni* = reference population size for the *i*th stratum, *ri* = rate in *i*th stratum, *ni* = cohort population size in *i*th stratum[12] (reference in main paper).

**3: Population projections for study region**

| **Age Group** | **2016** | **2025** | **2030** | **2035** | **2040** |
| --- | --- | --- | --- | --- | --- |
| **0 - 9** | 388044.3 | 382216.2 | 375329.1 | 371153.3 | 375857 |
| **10 19** | 351958 | 404972.4 | 410895.1 | 401769 | 395434.1 |
| **20 - 29** | 384027.4 | 350212.9 | 362547.7 | 394474.7 | 399026 |
| **30 - 39** | 394281.6 | 412934.2 | 396133 | 374661 | 386944.8 |
| **40 - 49** | 419179.7 | 404853.5 | 427446.9 | 429187.5 | 412559.5 |
| **50 - 59** | 422692.1 | 438061.7 | 412568.4 | 418391.9 | 441411.7 |
| **60 - 69** | 375553 | 417330.3 | 453611.2 | 445737.2 | 422812.8 |
| **70 - 79** | 270287.4 | 340554.7 | 343195.4 | 389069.9 | 424713 |
| **80 - 89** | 142941 | 180952.5 | 227774.5 | 244075 | 254071.9 |
| **90+** | 32282.69 | 41648.58 | 50575.83 | 64288.51 | 86471.94 |
| **Total** | 3181247 | 3373737 | 3460077 | 3532808 | 3599303 |

**Supplementary Table S4:** Projected populations of Bedfordshire, Cambridgeshire, Norfolk, Suffolk used in estimation of potential cases of operated chronic subdural haematoma. Data derived from ONS population data referenced in main article.

**4: R Code for the calculation of population projections**

###Population projections###

##Admin and package loading

Setwd(“…..”)

library(tidyverse)

library(DataCombine)

library(wesanderson)

library(epitools)

##Load data

tally<-read.csv(“csdh_cases_byage.csv”)

pop_proj<-read.csv("pop_projections.csv")

pop_proj<-pop_proj[1:10,]

pop_proj<-cbind(pop_proj, tally)

pop_proj<-pop_proj %>% mutate(totalpy=X2016*4) ##As covers 4 year period

pop_proj$n<-as.numeric(pop_proj$n)

##Poisson exact confidence intervals

ci<-pois.exact(pop_proj$n, pt=(pop_proj$totalpy), conf.level=0.95)

ci100k<-ci[,3:5]*100000

##Create dataframes for each year

pop_proj2016<-pop_proj %>% select(agegrp, X2016)

pop_proj2025<-pop_proj %>% select(agegrp, X2025)

pop_proj2030<-pop_proj %>% select(agegrp, X2030)

pop_proj2035<-pop_proj %>% select(agegrp, X2035)

pop_proj2040<-pop_proj %>% select(agegrp, X2040)

#rename the year columns

pop_proj2016<-pop_proj2016 %>% rename(popln=X2016) %>% mutate(year=2016)

pop_proj2025<-pop_proj2025 %>% rename(popln=X2025) %>% mutate(year=2025)

pop_proj2030<-pop_proj2030 %>% rename(popln=X2030) %>% mutate(year=2030)

pop_proj2035<-pop_proj2035 %>% rename(popln=X2035) %>% mutate(year=2035)

pop_proj2040<-pop_proj2040 %>% rename(popln=X2040) %>% mutate(year=2040)

##Calculate n of cases based on mid, upper, lower of the 95% CI

pop_proj2016<-pop_proj2016 %>% mutate(mid_n=popln*ci$rate) %>% mutate(low_n=popln*ci$lower) %>% mutate(upper_n=popln*ci$upper)

pop_proj2025<-pop_proj2025 %>% mutate(mid_n=popln*ci$rate) %>% mutate(low_n=popln*ci$lower) %>% mutate(upper_n=popln*ci$upper)

pop_proj2030<-pop_proj2030 %>% mutate(mid_n=popln*ci$rate) %>% mutate(low_n=popln*ci$lower) %>% mutate(upper_n=popln*ci$upper)

pop_proj2035<-pop_proj2035 %>% mutate(mid_n=popln*ci$rate) %>% mutate(low_n=popln*ci$lower) %>% mutate(upper_n=popln*ci$upper)

pop_proj2040<-pop_proj2040 %>% mutate(mid_n=popln*ci$rate) %>% mutate(low_n=popln*ci$lower) %>% mutate(upper_n=popln*ci$upper)

pop_proj_long<-rbind(pop_proj2016,pop_proj2025,pop_proj2030,pop_proj2035,pop_proj2040)

pop_proj_long

###Calculate total number of projected cases, and create separate dataframes for each year before binding together into final output (‘totals’)

totals<-data.frame(year=numeric(), mid=numeric(), lower=numeric(), upper=numeric())

total2016<-c(2016, sum(pop_proj2016$mid_n), sum(pop_proj2016$low_n), sum(pop_proj2016$upper_n))

total2025<-c(2025,sum(pop_proj2025$mid_n), sum(pop_proj2025$low_n), sum(pop_proj2025$upper_n))

total2030<-c(2030, sum(pop_proj2030$mid_n), sum(pop_proj2030$low_n), sum(pop_proj2030$upper_n))

total2035<-c(2035, sum(pop_proj2035$mid_n), sum(pop_proj2035$low_n), sum(pop_proj2035$upper_n))

total2040<-c(2040, sum(pop_proj2040$mid_n), sum(pop_proj2040$low_n), sum(pop_proj2040$upper_n))

totals[1,]<-total2016

totals[2,]<-total2025

totals[3,]<-total2030

totals[4,]<-total2035

totals[5,]<-total2040

totals<-totals %>% mutate(Region="Beds.,Cambs.,Norfolk,Suffolk")

totals_all<-totals

####This extrapolates case numbers to the whole region assuming that the proportion of cases coming from Beds.Cambs.Norfolk.Suffolk remains constant

totals_all<-totals_all %>% mutate(mid=mid*1.16) %>% mutate(lower=lower*1.16) %>% mutate(upper=upper*1.16)

totals_all<-totals_all %>% mutate(Region="EoE")

totals_long<-rbind(totals,totals_all)

**5: Estimated case numbers (generated from above code)**

| **Year** | **Mid** | **Lower** | **Upper** | **Region** |
| --- | --- | --- | --- | --- |
| **2016** | 111.5000 | 88.78003 | 142.3122 | **Beds. Cambs. Norfolk. Suffolk** |
| **2025** | 135.2592 | 108.46584 | 170.9356 | **Beds. Cambs. Norfolk. Suffolk** |
| **2030** | 150.3656 | 120.82324 | 189.3161 | **Beds. Cambs. Norfolk. Suffolk** |
| **2035** | 163.3816 | 131.36930 | 205.3318 | **Beds. Cambs. Norfolk. Suffolk** |
| **2040** | 174.0791 | 139.66439 | 219.1102 | **Beds. Cambs. Norfolk. Suffolk** |
| **2016** | 131.5700 | 104.76043 | 167.9284 | **EoE** |
| **2025** | 159.6059 | 127.98969 | 201.7041 | **EoE** |
| **2030** | 177.4314 | 142.57143 | 223.3930 | **EoE** |
| **2035** | 192.7903 | 155.01578 | 242.2916 | **EoE** |
| **2040** | 205.4133 | 164.80398 | 258.5500 | **EoE** |

**Supplementary Table S6:** Estimated numbers of cases of operated cSDH generated by R code in section 4 and reported in main paper. All projections were rounded to nearest whole numbers for reference in main text. Beds = Bedfordshire, Cambs = Cambridgeshire, EoE = East of England (extrapolated total workload assuming that proportion of cases from first four counties remains constant).
